# Influence of Gut Microbiota-Mediated Immune Regulation on Response to Chemotherapy

**DOI:** 10.3390/ph17050604

**Published:** 2024-05-08

**Authors:** Yufei Deng, Xiaoying Hou, Haiping Wang, Hongzhi Du, Yuchen Liu

**Affiliations:** 1 Wuhan Institute of Biomedical Sciences, School of Medicine, Jianghan University, Wuhan 430056, China; grys00797@126.com (Y.D.); jhhxy2021@jhun.edu.cn (X.H.); wang-haiping@jhun.edu.cn (H.W.); 2 Cancer Institute, School of Medicine, Jianghan University, Wuhan 430056, China; 3 Hubei Key Laboratory of Cognitive and Affective Disorders, Jianghan University, Wuhan 430056, China; 4 School of Pharmacy, Hubei University of Chinese Medicine, Wuhan 430065, China

**Keywords:** gut microbiota, immune regulation, chemotherapy response

## Abstract

The involvement of the gut microbiota in anti-cancer treatment has gained increasing attention. Alterations to the structure and function of the gut bacteria are important factors in the development of cancer as well as the efficacy of chemotherapy. Recent studies have confirmed that the gut microbiota and related metabolites influence the pharmacological activity of chemotherapeutic agents through interactions with the immune system. This review aims to summarize the current knowledge of how malignant tumor and chemotherapy affect the gut microbiota, how the gut microbiota regulates host immune response, and how interactions between the gut microbiota and host immune response influence the efficacy of chemotherapy. Recent advances in strategies for increasing the efficiency of chemotherapy based on the gut microbiota are also described. Deciphering the complex homeostasis maintained by the gut microbiota and host immunity provides a solid scientific basis for bacterial intervention in chemotherapy.

## 1. Introduction

Exciting development in the field of cancer treatment has been witnessed over the past 50 years. Various methods, such as surgery, chemotherapy, radiotherapy, immunotherapy, and molecular targeted therapy are efficient when properly employed in anti-cancer procedures [1]. Chemotherapy, in particular, is effective in controlling the progression of cancer and relieving related symptoms. Due to its efficiency in different malignant tumors and accessible cost, chemotherapy is now the recommended first-line treatment for cancer. However, the non-selective cytotoxicity of chemotherapy may lead to serious side-effects, such as acute kidney injury and gastrointestinal symptoms. Chemotherapy may become less efficient with the widespread occurrence of primary resistance and rapidly generated acquired resistance. Therefore, novel treatment strategies to reduce the associated toxicity and increase the efficacy of chemotherapy should be developed.

**Figure 1:**
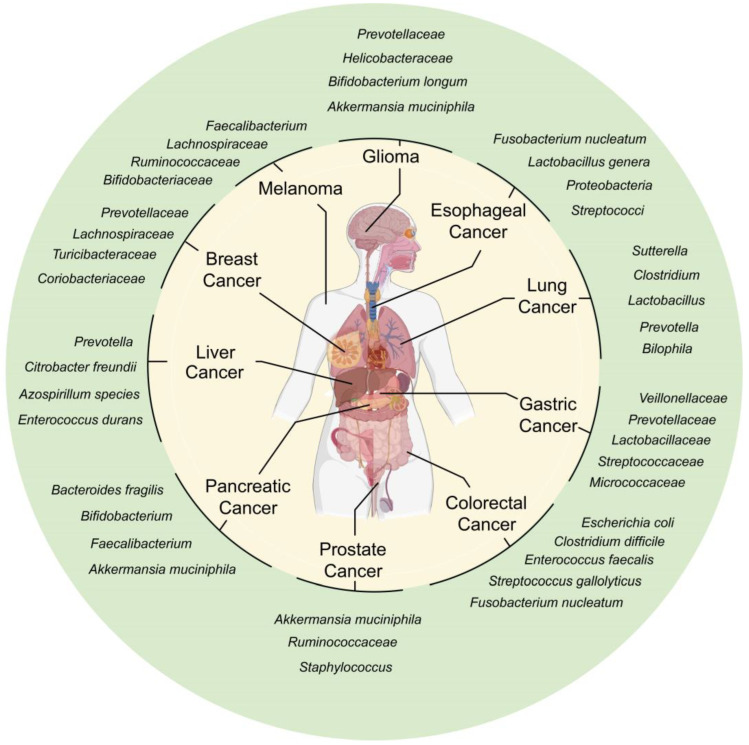
Effect of gut microbiota on different cancers. Specific gut microbiota affect the initiation, progression, diagnosis, treatment, prognosis, and prediction of a variety of cancers.

One important recent finding is that the gut microbiota are involved in many physiological activities as well as the development of various diseases. More than 10^13^ bacteria exist in the gastrointestinal tract (GIT) [2], mainly composed of *Firmicutes*, *Bacteroidetes*, *Actinobacteria*, *Proteobacteria*, and *Verrucomicrobia* [3,4,5]. It is known that gut microbiota are involved in many aspects of cancer, such as its initiation, progression, diagnosis, treatment, and prognosis [6] (Figure 1). In addition, growing evidence has shown that the gut microbiota also regulate the efficacy and toxicity of chemotherapy through drug metabolization and immune response [7]. It is envisaged that gut microbiota-based interventions combined with chemotherapy might improve the outcome of cancer treatments.

In this review, we discuss the interactions among chemotherapy, the gut microbiota, and the host immune response. We describe the current knowledge on how malignant tumors and chemotherapy affect the gut microbiota, how the gut microbiota regulate the host immune response, and how interactions between the gut microbiota and host immune response influence the efficacy of chemotherapy. We also summarize recent advances in strategies for increasing the efficiency of chemotherapy based on the gut microbiota.

## 2. Current Clinical Applications of Chemotherapy

Chemotherapy is a classic anti-cancer treatment, which has certain advantages in terms of controlling the progression of cancer when compared with local treatment. For example, neoadjuvant chemotherapy transforms inoperable breast cancer into an operable state, thus effectively improving its prognosis [8]. 5-fluorouracil (5-FU) is the standard post-operative chemotherapy for colorectal cancer (CRC), which significantly reduces the risk of local recurrence in patients [9].

Although extensively applied in anti-cancer treatments [10], chemotherapy has limitations that significantly decrease its efficacy, including poor bio-availability, rapid blood clearance, non-selective distribution, and drug resistance [11,12]. A retrospective study found that the objective response (OR) rate of platinum-based drugs in CRC patients was less than 45% [13], while the pathological complete response (PCR) rate in breast cancer patients was less than 25% [14]. The non-selective cytotoxic effects of chemotherapeutic drugs can also lead to multiple side effects, including short- and long-term treatment-related adverse events (AEs) such as weight loss, marrow suppression, skeletal muscle loss, psychiatric complications, and a variety of gastrointestinal symptoms [15,16]. A clinical study found that nearly 60% of the patients with metastatic CRC treated with FOLFOX experienced weight loss [17]. Meanwhile, diarrhea occurred in 50–80% of cancer patients after chemotherapy using 5-FU, capecitabine, irinotecan, and others [2,18,19].

Chemotherapy also injures the intestinal mucosal barrier and intestinal homeostasis, causing dysbiosis of the gut microbiota and mucosal inflammation [4,15]. For example, cisplatin impairs the integrity of the intestinal epithelial barrier and promotes microbial relocation through the circulatory system, therefore leading to systemic inflammation. Administration of 5-FU increases the presence of Gram-negative bacteria such as *Bacteroides* in the gut, which induces inflammation through the nuclear factor kappa B (NF-κB) pathway [4,20,21]. The use of ephedrine successfully reversed gut microbiota dysregulation, inhibited inflammation, and restored intestinal barrier function in 5-FU-induced intestinal mucositis (FUIIM) model mice [22].

Dose-limiting toxicity (DLT) is another important factor that has an impact on the outcome of chemotherapy [23]. According to the requirements of the National Cancer Institute (NCI) for recording AEs in cancer trials, the significate toxic effect observed with different doses is defined as the DLT, and the maximum tolerated dose (MTD) in chemotherapy is determined based on the DLT. For example, hematological toxicity and nausea are considered as common DLTs of gemcitabine. In a clinical trial, gemcitabine given to patients increased gradually until one-third of the patients developed hematological toxicity or nausea; the corresponding dosage was recorded as the MTD of gemcitabine [24]. Similarly, thrombocytopenic fever is the known DLT for paclitaxel; for example, when using paclitaxel to treat metastatic breast cancer, thrombocytopenic fever is the sign of reaching the MTD, and no further increase in dosage should be applied [25]. It is known that the response to chemotherapy is closely related to the used dose, and a 20% dose reduction may lead to a 50% decrease in the cure rate [26]. As DLT determines the MTD of chemotherapy directly while affecting the efficacy of chemotherapy indirectly, it is difficult to find a balance to improve the outcome of anti-cancer treatment.

## 3. Gut Microbiota and Chemotherapy

### 3.1. Gut Microbiota in Tumorigenesis and Development

The gut microbiota are described as the second-largest organ of the human body, which is connected with physiological and pathological processes through supporting energy metabolism, nutrient catabolism and absorption, the synthesis of vitamins and essential amino acids, maintaining intestinal mucosal barrier integrity, inactivation of toxins and carcinogens, immunomodulation, and protection from pathogens [27,28]. Multiple internal and external factors can modify the composition and function of the gut microbiota as well as related metabolites, which may affect the occurrence and development of cancer through DNA damage in the host, the production of oncogenic/anti-cancer metabolites, activation of oncogenic signaling pathways, immune cell infiltration, and secretion of inflammatory factors [7,29].

The gut microbiota are involved in a complex system of catabolism and anabolism, in which various metabolites are derived and diffuse into the circulatory system to affect general homeostasis. Carcinogenesis is known to be promoted by toxic or carcinogenic metabolites such as amines and sulfide, which are frequent products of gut microbiota metabolization [7]. Trimethylamine (TMA) and its metabolite trimethylamine amine N-oxide (TMAO), for instance, are generated by the gut microbiota and promote the development of hepatocellular carcinoma (HCC) [30]. Hydrogen sulfide produced by the gut microbiota has been found to be related to the occurrence and development of CRC [31], although the underlying mechanism requires further elucidation. Moreover, the distribution and function of the gut microbiota are related to complications in cancer patients, such as fever [32], tumor-related fatigue [33], cancerous pain [34], and venous thrombosis [35]. Studies have shown that the increased level of *Akkermansia muciniphila* in cancer patients could damage the intestinal mucus layer and induce fever, and the use of antibiotics or bacteria-derived propionate prevented these complications in animal models [32].

Gut microbes play an important role in oncogenesis through the biotransformation of steroid hormones. *Clostridium leptum* and *Clostridium coccoides* produce β-glucuronidase and upregulate the biotransformation of active estrogen, which then promotes the development of breast cancer [3,36]. On the other hand, some bacteria in the GIT have functions which may alleviate tumors; for example, *Lactiplantibacillus plantarum*-12 secretes exopolysaccharides that alleviated AOM/DSS-induced colon cancer symptoms in mice [37].

Inflammation is often associated with the development and progression of cancer. The gut microbiota affect tumor development through interactions with the host immune system. Gut microbes regulate inflammatory factors such as tumor necrosis factor-α (TNF-α), interleukin-1 (IL-1), and NF-κB [38,39,40] to activate or suppress cancer-related inflammation. Zhong et al. found that dysbiosis of the gut microbiota increased IL-6 levels in both the tumor and serum through activation of the NF-κB signal pathway, thus promoting the malignant progression of prostate cancer [41]. Moreover, the gut microbiota interact with the host’s immune system through bacterial translocation. Under pathological conditions, gut barrier disruption and dysbiosis promote the transfer of bacteria and their by-products into the portal vein, liver, and other off-site locations, inducing inflammatory responses and, therefore, influencing the development of tumors [42,43]; more specific content in this line is discussed in Section 4.3.

The gut microbiome and related metabolites may also serve as biomarkers for early diagnosis of lung, liver cancer and CRC [44,45]. For example, the significant and simultaneous enrichment of *B. fragilis*, *Porphyromonas asaccharolytica*, *Parvimonas micra*, *Prevotella intermedia*, *Alistipes finegoldii*, and *Thermanaerovibrio acidaminovorans* has been reported as a diagnostic marker for CRC [46]. Another study found that *Ruminococcus*, *Enterobacteriaceae*, and *Lachnospiraceae* were highly enriched in lung cancer patients, while *Faecalibacterium*, *Streptococcus*, *Bifidobacterium*, and *Veillonella* presented higher levels in healthy people [47]. *Alistipes*, *Phascolarctobacterium*, and *Ruminococcus* have been shown to be significantly decreased in early HCC, while *Klebsiella* and *Haemophilus* were increased [48]. Moreover, a study has shown that the decreased abundance of *Firmicutes* and *Actinomycetes*, together with significantly increased levels of *Clostridium*, *Bacteroides*, and *Proteus*, affect the pancreatic microenvironment and contribute to the development of pancreatic cancer in smokers [49]. Therefore, the altered abundances of specific bacteria may serve as predictive biomarkers for cancer diagnosis and prognosis.

### 3.2. Interaction between Gut Microbiota and Chemotherapy

Chemotherapy alters the gut microbiota on multiple levels. First, chemotherapy changes the diversity and species richness of the gut microbiota [50,51]. It has been proven that most chemotherapies lead to decreases in *Lactobacillus*, *Bacteroides* [52], *Bifidobacterium*, and *Enterococcus* [2,53,54], as well as an increase in *Firmicutes*, *Escherichia coli*, and *Staphylococcus* [40,44,55]. Cyclophosphamide (CTX) decreased the abundance of intestinal *Bifidobacterium* while increasing the levels of *Proteus* and *E. coli* [16] in CRC patients. Irinotecan, on the other hand, significantly increased the levels of *Clostridium cecal* and *Enterobacteriaceae* in the intestines of CRC-bearing rats [44]. Second, chemotherapy affects the gut microbiota through damaging the mucosal barrier of the GIT [36]. The most important structure of the mucosal barrier consists of complete intestinal mucosal epithelial cells and the tight junctions between epithelial cells [56]. This mechanical barrier can effectively prevent harmful bacteria and endotoxins from entering the bloodstream through the intestinal mucosa [57]. Studies have shown that chemotherapy could damage epithelial cells to directly break the mucosal barrier as well as damage intestinal stem cells to indirectly impair mucosal barrier function. Increased permeability of the mucosal barrier allows intestinal bacteria to move to the spleen, liver, peritoneal cavity, and blood. The translocated bacteria may interact with immune system and induce inflammation, which subsequently affects the efficacy of chemotherapy [58]. For example, chemotherapy with CTX was shown to damage the intestinal epithelium and increase the permeability of the intestinal mucosal barrier. The following translocation of *Enterococcus hirae* and *Lactobacillus Johansonii* to tumor-draining lymph nodes (TDLNs) induced differentiation of CD4^+^T-cells to Th17 in the tumor microenvironment (TME), thus enhancing the anti-fibrosarcoma efficacy of CTX in vivo [59]. Oxaliplatin also impairs gastrointestinal barrier function through reactive oxygen species (ROS)-induced DNA damage [3], which leads to translocation of microbiota, consequently affecting immune homeostasis and chemotherapy sensitivity [60]. Therefore, chemotherapy has multiple effects on the gut microbiota, which may further affect the development of cancer and the response to chemotherapy.

The gut microbiota are involved in the response to chemotherapy. Gut microbiota promoted the ability of tumor-infiltrating hematopoietic cells to release ROS, enhancing the efficacy of oxaliplatin against lymphoma or colon cancer in mice [61,62,63]. After translocation to the secondary lymph organs, *E. hirae* and *Barnesiella intestinihominis* enhanced the anti-tumor effect of CTX through regulating Th1 responses [59]. The gut microbiota also regulate the efficacy of chemotherapy through their metabolites. Butyrate is a metabolite of gut microbiota species such as *Eubacterium rectale* and *Faecalibacterium prausnitzii* [64]. It is known to increase the anti-tumor activity of irinotecan against colon cancer cell lines [65] through reducing the expression of the drug resistance-related protein P-glycoprotein. Furthermore, secondary bile acids—such as ursodeoxycholic acid (UDCA) and ursocholic acid (UCA)—are associated with improved objective responses (OR) in patients with HCC receiving treatment with immune checkpoint inhibitors (ICIs) [66]. A study previously published by our group also proved that the *Prevotella*-related metabolite 3-Oxocholic acid decreased the efficacy of FOLFOX against CRC in vitro [67]. Chemotherapy with cisplatin can result in severe toxic side effects, such as weight loss and intestinal toxicity, which limits its efficacy and wider application. Studies have shown that oral supplementation of *Lactobacillus* increased the expression of IFN-γ, GZMB, and PFR1 in CD8^+^T-cells, which improved the efficacy of cisplatin against Lewis lung cancer [68] and reduced its toxic side effects [69] in mice. Gemcitabine is commonly used in chemotherapy for pancreatic ductal adenocarcinoma (PDAC), despite the rapid development of drug resistance [70]. Recent studies have found that the antibiotic ciprofloxacin successfully reversed gemcitabine chemotherapy resistance in mice [71], suggesting a correlation between gemcitabine resistance and the gut microbiota.

In addition, the gut microbiota are involved in metabolizing chemotherapeutic drugs into active, inactive, or toxic forms. The efficacy of more than 60 chemotherapeutic drugs is altered after this transformation [2,72,73]. For example, glucuronidated SN38 (SN38-G) is the active metabolite of irinotecan. Intestinal bacteria produce β-glucuronidase to convert intestinal SN38-G into toxic SN38, which severely reduces the anti-CRC effect of irinotecan [19,74,75]. Meanwhile, toxic SN38 induces gastrointestinal reactions such as diarrhea and intestinal damage [44]. In addition, cytidine deaminase produced by *Mycoplasma hydrohinis* can metabolize Gemcitabine into the inactive product 2′,2′-difluoro-2′-deoxyuridine, which reduces the drug activity by more than 10-fold [76].

**Figure 2:**
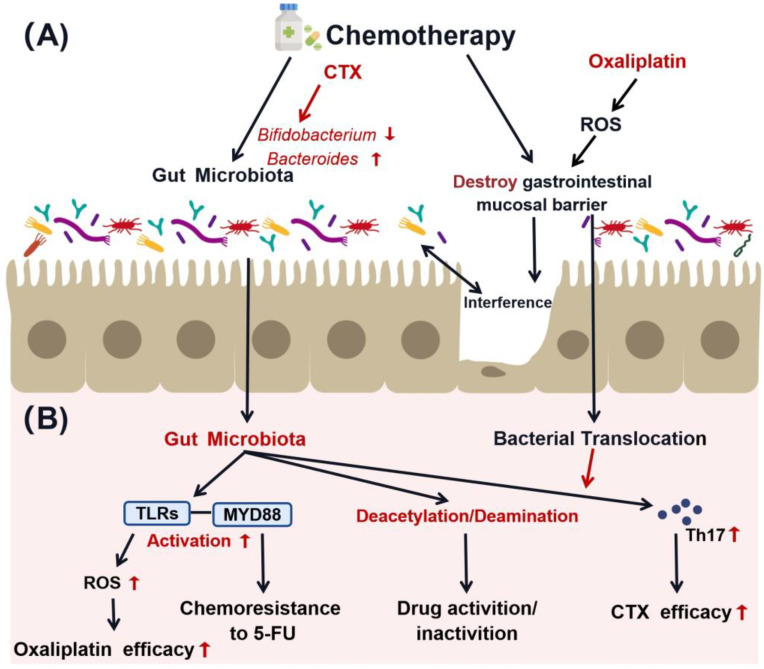
Interaction between gut microbiota and chemotherapy. (**A**) Chemotherapy affects gut microbiota colonization directly through influencing their abundance/diversity or indirectly through destroying the intestinal mucosal barrier. (**B**) The gut microbiota can influence the pharmacological effect or biotransformation of chemotherapeutic drugs through biochemical reactions including deamination and deacetylation. CTX, Cyclophosphamide. 5-FU, 5-Fluorouracil. ROS, reactive oxygen species.

In summary, the interaction between chemotherapy and gut microbes can alter the distribution and function of the gut microbiota, thus modulating the efficacy and toxicity of the chemotherapeutic agents directly through drug biotransformation or indirectly through the host immune system. Chemotherapy also regulates the colonization of gut microbiota. These interactions affect the outcome of anti-cancer treatments [3] and the prognosis of patients (Figure 2).

## 4. Gut Microbiota-Mediated Immunomodulation in Chemotherapy Sensitivity

### 4.1. Effects of Immune Modulation on Chemotherapy Response

The GIT is considered to be the largest immune organ in the human body, with about 70% of all lymphocytes residing in the intestinal epithelium, intestinal lamina propria, and intestinal draining mesenteric lymph nodes. Lymphocytes in the GIT contain a large number of activated T-cells, antibody secretion plasma cells, and innate immune cells [77]. Tumor immune escape is one of the most typical characteristics of cancer cells during tumorigenesis and development, the underlying mechanisms of which include decreased immune detection ability, transition to an immuno-suppressive microenvironment, upregulation of immune checkpoint proteins such as PD-L1, and decreased activity of CD8^+^T-cells [78]. Inhibition of immune cells in the TME is essential for tumor immune escape and resistance to anti-tumor therapy. As has been previously reported, the gut microbiota can inhibit immune cells in the TME to promote tumor immune escape. For example, the outer membrane protein Fab2 of *Fusobacterium nucleatum* binds to the inhibitory T-cell immunoglobulin and ITIM domain (TIGIT) receptors in tumor-infiltrating NK cells and T-cells, then decreases the anti-cancer effects of these immune cells [79]. With derived deoxycholic acid, *Clostridium scindens* targets the cell membrane calcium pump plasma membrane Ca^2+^ATPase (PMCA) to promote Ca^2+^ efflux, which leads to a reduction in intracellular Ca^2+^ and inhibition of NFAT2, thus decreasing the anti-cancer function of effector CD8^+^T-cells [80], which ultimately promotes cancer immune escape. On the other hand, gut bacteria enhance immune surveillance and inhibit the development of cancers. Wang et al. found that the intestinal bacteria *Lactobacillus johnsonii* can transform tryptophan into 3-Indole propionic acid (IPA), which contributes to the infiltration of CD8^+^T-cells and cytokine IFN-γ into tumors, thus enhancing the efficacy of ICI in colon cancer-bearing mice [81].

Immune regulation is critical in chemotherapy response. For instance, the level of infiltration of CD8^+^T-cells and T follicular helper (Tfh) cells into osteosarcoma tissue was significantly correlated with a positive chemotherapy response [82]. Zhou et al. have revealed that the activation of the p300/CBP pathway and NF-κB can be used to predict the anti-tumor effect of oxaliplatin against colon cancer cells [83]. Moreover, the combination of a PD-1/PD-L1 inhibitor and chemotherapeutic agents such as cisplatin significantly increased the prognosis of extensive-stage small-cell lung cancer (ES-SCLC) without raising AEs [84].

Alternatively, some chemotherapy approaches stimulate the secretion of cytokines, thereby exerting an immunomodulatory effect. A study has found that 5-FU administration significantly increased inflammatory cytokines such as TNF-α and NOS2 [85] in CRC mice. The administration of methotrexate (MTX) also upregulated intestinal IL-1β, TNF-α, and IFN-γ in mice [50].

### 4.2. Interaction of Gut Microbiota and Related Metabolites with Immune System

Intestinal homeostasis can be maintained by the gut microbiota. The gut microbiota occupy ecological niches to prevent pathogenic micro-organisms from binding with epithelial cells and inducing immune responses [3,43]. On the other hand, the gut microbiome is closely related to immune responses [44]; for example, the gut microbiota can promote the production of cytokines such as IL-22, IL-17, and IL-10 [77,86], in order to regulate the host immune system. Cytokines also activate primary/secondary lymphoid organs to regulate the tumor immune microenvironment [87]. Moreover, the gut microbiota contribute to the development of intestinal epithelial cells (IEC). Studies have shown that lipopolysaccharides (LPS) and flagella proteins of the gut microbiota promote IEC proliferation [88]. These components also act as ligands and bind with the NOD domain-like receptors (NLRs) and Toll-like receptors (TLRs) that are expressed by IEC, consequently regulating the immune barrier function of IEC [49,79,89,90]. Flagellin promotes the production of IL-8 through binding to NLRs and TLRs, while LPS can induce the secretion of mucin 2 (MUC2) from colon goblet cells [88]. LPS also promotes the secretion of pro-inflammatory cytokines such as TNF-α, which plays an important role in the activation of macrophages, as well as the generation and clearance of inflammation [91].

Abnormal gut microbiota can have negative impacts on the homeostasis of the immune system. The study of Li et al. showed that dysfunction of the gut microbiota leads to abnormal hyperplasia of intestinal epithelial tuft cells and enhances the secretion of IL-25, which induces M2 macrophage polarization [92]. Dysbiosis induced by fluconazole led to decreased expression levels of IL-1β, IL-6, and IL-8, as well as increased expression of IL-2, LZM, and IgM, suggesting that the gut microbiota interfere with immune responses [93].

**Figure 3:**
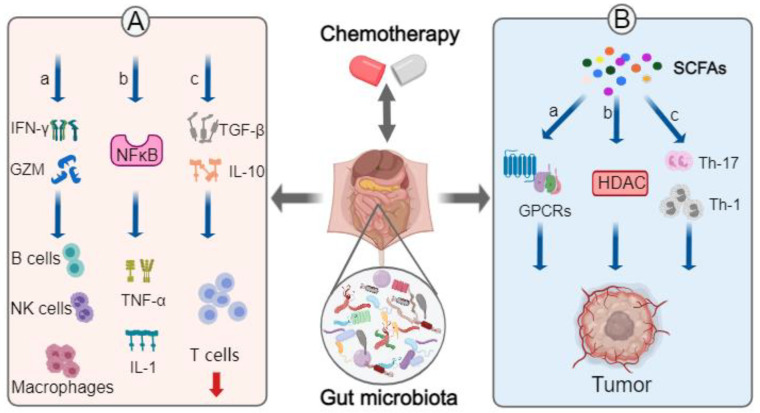
Gut microbiota-mediated immune response to chemotherapy—Direct (**A**) and Indirect (**B**) impacts. (**A**) a. Gut microbiota affect macrophage NK cells and B cells through regulating the production of IFN-γ and GZM; b. Gut microbiota can promote the secretion of pro-inflammatory cytokines such as TNF-α or IL-1 through activation of NF-κB; c. Gut microbiota reduce the level of Treg cells via IL-10 and TGF-β. (**B**) The gut microbiota-related metabolites SCFAs affect the overall anti-tumor function through activating GPCRs (a), inhibiting HDAC (b), or influencing TH1 and TH17 cell maturation (c). GZM, granzymes. NK cell, natural killer cell. SCFAs, short-chain fatty acids. HDAC, histone deacetylase. GPCRs, G-protein-coupled receptors.

The gut microbiota regulate the infiltration and activation of immune cells, as well as the secretion of inflammatory factors, to affect intestinal homeostasis. The gut microbiota participate in reducing the pro-inflammatory response through regulating the production of IL-10 by Tregs cells and transforming growth factor beta (TGF-β). *Bacteroides fragilis*, for instance, upregulates the secretion of anti-inflammatory cytokine IL-10 [94]. Meanwhile, IL-10 knockout increased the pathogenicity of *Bacteroidaceae*, *Porphyromonadaceae*, *Bacteroides ovatus*, and *Bacteroides acidifaciens*, and increased the level of Th17 and Treg cells [95]. The gut microbiota can increase the expression of IFN-γ and granzymes by helper (CD4^+^) and cytotoxic (CD8^+^) T-cells, which promotes the recruitment of macrophages, as well as the activation and maintenance of natural killer (NK) cells, lymphocytes, B cells, cytotoxic, and helper T lymphocytes [28,96]. Meanwhile, the gut microbiota also induces the activation of NF-κB, promoting the secretion of pro-inflammatory cytokines such as TNF-α and IL-1 to modulate the circulating immunity of the host (Figure 3A) [46,54,94,97].

Furthermore, gut microbiota-related metabolites affect the systemic immune response. Intestinal bacteria-related metabolites, such as short-chain fatty acids (SCFAs) [98], bile acid [99], tryptophan metabolites [100], and methionine [101], are absorbed into the circulatory system. They then bind with cell surface receptors to regulate the differentiation and function of immune cells [102]. For example, SCFAs derived from *Clostridium butyricum*, *Bifidobacterium*, *Lactobacillus rhamnosus*, *Streptococcus thermophilus*, *Lactobacillus reuteri*, *Lactobacillus casei*, and *Lactobacillus acidophilus* [103] are involved in activation of G-protein-coupled receptors, which are widely present in the immune system. SCFAs also promote the differentiation of T-cells into effector T-cells (Th1 and Th17 cell) [94,104]. Furthermore, derived SCFAs inhibit histone deacetylases (HDACs), which have many implications in the innate immune process through regulating the Toll-like receptor and interferon signaling pathway. Meanwhile, HDACs regulate the antigen presentation process, lymphocyte growth, differentiation, and polarization in the adaptive immune process. Research from the Harvard School of Public Health has suggested that SCFAs can prevent colitis in mice through Regulatory T-cells (Tregs) [105]. Another example is gut microbiota-derived D-methionine: it has been found that D-methionine relieves cisplatin-induced intestinal mucositis through increasing the abundance of beneficial micro-organisms such as *Lactobacillus*, which reduced oxidative stress and pro-inflammatory immune responses [106]. In addition, the gut microbiota convert primary bile acids into secondary bile acids [107], which are known to suppress the expression of chemokines (e.g., CXCLs) as well as the accumulation of NKT cells in the liver, which promoted liver cancer development in mice [99]. Taken together, these results suggest that gut bacteria play essential roles in immune regulation.

### 4.3. Interactions among Gut Microbiota, Immune System, and Chemotherapy

Interactions between the gut microbiota and the immune system may influence the activity and toxicity of chemotherapeutics through the host immune response, inflammatory, and immuno-suppressive pathways [94], while chemotherapy could alter the distribution and function of the gut microbiota. These changes induce immune responses which, in turn, influence the activity of chemotherapeutic agents. Thus, maintaining the balance of the gut microbiota may reduce the inflammation induced by chemotherapy [108].

As previously described, bacteria can translocate from the gut to secondary lymphoid organs, which activates an immune response and induces inflammation in distal organs. Studies confirmed that CTX—a classic alkylating agent—promoted the translocation of gut bacteria to the intestinal lamina propria [109], then increased the differentiation of Th17 cells through the MyD88 signaling pathway [110,111]. The use of antibiotics significantly decreased the anti-tumor effect of CTX, indicating that the gut microbiota are a key factor affecting the efficacy of CTX.

Both inherent and exogenous gut microbiota can interact with immune system and, thus, play roles in the host’s sensitivity to chemotherapy; in particular, the inherent gut microbiota-mediated immune response influences chemotherapy sensitivity. Gut microbiota species such as *B. fragilis* can release *B. fragilis* toxin, which upregulates the expression of spermine oxidase (SMO) in colonic epithelial cells [112,113]. *B. fragilis* also increases the levels of *Nox1* and *Cybb* encoding ROS-generating NADPH oxidase 2 (NOX2) [114] and stimulates tumor-infiltrating myeloid cells to produce ROS. High levels of ROS result in oxidative stress, leading to DNA damage and apoptosis, which affect the anti-tumor efficacy of oxaliplatin, alkylating agents, anthracycline, pavonlotoxin, camptothecin, and so on [38,115]. Studies have shown that the gut microbiota could stimulate tumor-infiltrating myeloid cells to produce ROS through the TLR4-MYD88 signaling pathway, which increased the anti-tumor efficacy of oxaliplatin in mice bearing colon cancer. In contrast, elimination of the gut microbiota with antibiotics reduced the sensitivity of the tumor to oxaliplatin [27,116]. Our previous study also revealed that the *Prevotella*-derived metabolite 3-Oxocholic acid significantly increased the expression levels of IL-1β and TNF-α, thus decreasing the efficacy of FOLFOX against CRC in vitro [67]. Another example is 5-FU, a first-line chemotherapeutic agent for various gastrointestinal malignant tumors. Studies have confirmed that *F. nucleatum* activated autophagy through the TLR4-MYD88 signaling pathway, resulting in chemo-resistance to 5-FU [117] and Oxaliplatin [118].

On the other hand, exogenous microbiota interventions such as ingestion of probiotics affect the response to chemotherapy through adjusting immune regulation and the inflammatory response. Probiotics also interact with the gut microbiota and their derived metabolites (e.g., SCFAs, bile acids) to regulate carcinogenesis (Figure 3B). A study on probiotics found that *Lactobacillus paracasei* and *L. reuteri* in combination with gemcitabine could enhance the anti-cancer effect and alleviate liver toxicity in PDAC, when compared to gemcitabine used without probiotics [119]. *L. paracasei* has also been shown to decrease the IL-6 level in mice, which may contribute to inhibition of PDAC [119]. It is known that chemotherapy induces atrophy of the gastrointestinal villi and disruption of the mucosal barrier through increasing the levels of matrix metalloproteinase (MMP-9), NF-κB, IL-1β, and TNF-α in the gastrointestinal mucosa [4,50,53]. Another study found that *Lactobacillus fermentum* together with the prebiotic fructooligosaccharide alleviated 5-FU-induced intestinal mucositis and improved intestinal barrier function [106].

In conclusion, gut microbiota-mediated immune responses play important roles in the development of cancers and sensitivity to chemotherapy (Table 1). Therefore, additional gut microbiota interventions may be a beneficial strategy for enhancing the efficacy of chemotherapy in anti-cancer treatments.

## 5. Clinical Application

**Figure 4:**
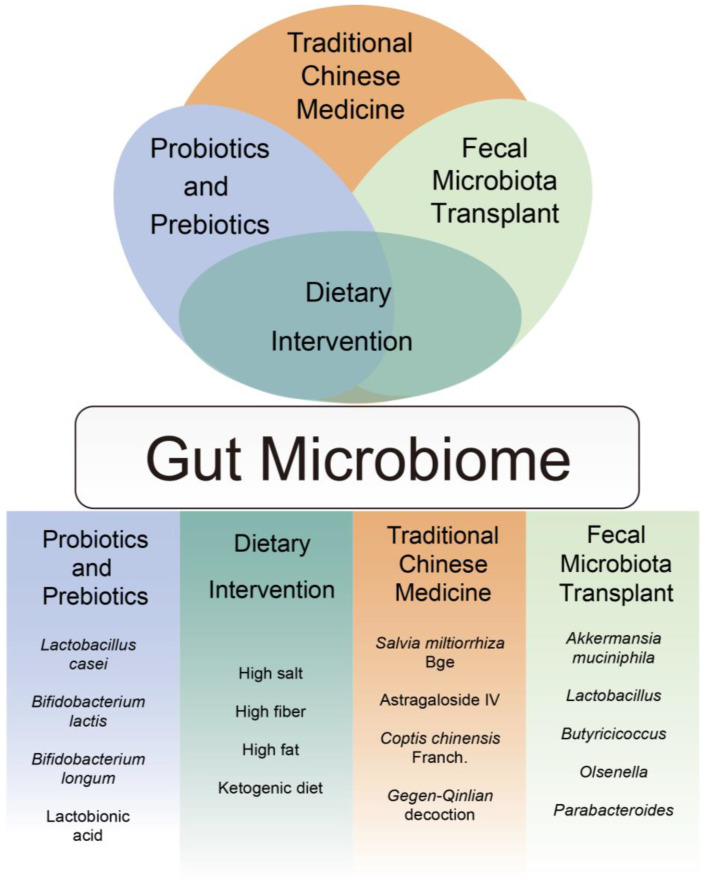
Clinical application of gut microbiota. The figure lists the intestinal microbiome interventions that have been applied or are in progress. Probiotics/Prebiotics: An elevated level of *Lactobacillus casei*, *Bifidobacterium lactis*, and *Bifidobacterium longum* is associated with reduced chemotherapy toxicity. Lactobionic acid serves as a carrier to enhance the inhibitory effect of doxorubicin against liver cancer in vitro. Dietary intervention: a high-salt diet and a high-fiber diet inhibit the development of colon cancer in mice, whereas a high-fat diet promotes cancer development. The ketogenic diet enhances anti-tumor effect of PD-1 in melanoma mice. Traditional Chinese Medicine: *Salvia miltiorrhiza* Bge increases the anti-tumor effect of PD-1 in mice with liver cancer. *Coptis chinensis* Franch significantly inhibits the growth of gastric cancer in mice. *Astragalus membranaceus* inhibits the development of colon cancer, while Gegen Qinlian Tang (GQD) enhances the anti-tumor effect of PD-1 in CRC mice. Fecal Microbiota Transplant: *Akkermansia muciniphila* enhances the efficacy of CDDP in mice with Lewis lung cancer. *Butyricicoccus* and *Parabacteroides* alleviate colorectal cancer. *Lactobacillus* and *Olsenella* increase the anti-tumor efficacy of immune checkpoint blockade in mice with colon cancer.

The interactions between the gut microbiota or their derived metabolites with the immune system mediate the pharmacological efficacy of chemotherapeutic agents. This interaction is also a potential target for reducing the toxicity and enhancing the efficacy of chemotherapy. To date, widely investigated gut microbiota interventions include ingestion of probiotics/prebiotics, dietary interventions, Traditional Chinese Medicine (TCM), and Fecal Microbiota Transplant (FMT). The positive results reported in biomedical research offer greater opportunities for their future application in anti-cancer treatments (Figure 4).

Probiotics/prebiotics are known to modulate the gut microbiota and related metabolites in cancer patients, which may interfere with the response to chemotherapy. A clinical study has confirmed that the probiotics *Bifidobacterium lactis* Bl-04 and *L. acidophilus* NCFM increased the intestinal production of butyrate by *Faecalibacterium* and *Clostridiales* in CRC patients [122]. Other studies have shown that probiotics could alleviate the adverse effect of chemotherapy in patients through modulation of the gut microbiota. Post-operative probiotic administration with combined *Bifidobacterium infants*, *Lactobacillus acidophilus*, *Enterococcus faecalis*, and *Bacillus cereus* tablets reduced chemotherapy-induced gastrointestinal complications and gut microbiota imbalances in CRC patients [123]. Administration of these probiotics effectively restored the gut microbe balance, with mild increases in *Bifidobacterium*, *Streptococcus*, and *Blautia*. Furthermore, probiotics could also promote the production of SCFAs, particularly increasing acetate, butyrate, and propionate. Another study has found that probiotic supplementation with *Bifidobacterium longum*, *L. acidophilus*, and *E. faecalis* reduced chemotherapy-related cognitive impairment in breast cancer patients [124]. These effects demonstrate that probiotics/prebiotics regulate the response to chemotherapy through modulation of the gut microbiota. Furthermore, intervention with probiotics regulates the response to chemotherapy through gut microbiota-related inflammatory factors. The probiotic *Lactobacillus rhamnosus* (Lcr35) reversed the dysbiosis of gut microbiota after treatment with FOLFOX and down-regulated inflammatory factors such as IL-6 and TNF-α in the intestine, thus alleviating intestinal damage [125]. Meanwhile, it is known that *B. longum* can alleviate irinotecan-induced intestinal dysbiosis through reducing the levels of IL-1β and IL-18 and re-establishing the intestinal mucosal barrier [126]. The study of Chen et al. has indicated that probiotic VSL#3 (a probiotic mixture containing eight strains of probiotic bacteria) increased the abundance of bacterial-derived propionate and butyrate. These metabolites increase the expression of CCL20, which upregulates the recruitment of Th17 cells in lung endothelial cells, thus inhibiting the lung metastasis of melanoma [127]. Moreover, probiotics/prebiotics interfere with efficiency of anti-CRC treatment by affecting the colonization of pathogenic bacteria such as *Clostridium difficile* and *Staphylococcus aureus* in the intestine. Probiotics/prebiotics also act by regulating intestinal immunity and affecting the production of SCFAs [128]. However, the beneficial effects of probiotics/prebiotics on the human body are complicated by individual differences, and the underlying mechanisms still require further elucidation [128].

Dietary interventions have been found to be closely related to tumorigenesis, development, and cancer therapy. Considering the current status of pre-clinical research, most investigations focused on dietary interventions have been carried out in rodent models. The study of Meynet et al. showed that dietary restriction (DR) with 70% of the normal quantity improved the sensitivity to BH3 mimetic therapy in mice with lymphomas [129]. On the other hand, a high-fat diet (HFD) increased the pathogenic bacteria *Alistipes sp. Marseille P5997* and *Alistipes sp. 5CPEGH6* in mice and promoted the carcinogenesis of colon cancer [130]. Further investigation revealed that HFD upregulated the synthesis of Glycerophospholipid and increased the carcinogenic metabolite LPA in mice. Studies have also found that DR changed the composition of the gut microbiota in mice and reduced the expression levels of the inflammatory factors IL-10, IL-1β, TNF-α, IFN-γ, and IL-6, thus relieving the MTX-induced enteric dysfunction [131]. Therefore, diet is an important factor in modulating the composition of the gut microbiota, the production of metabolites by the gut microbiota, and intestinal inflammation [132]. Due to the diversity of diets and the complexity of the inner environment of individuals, the impact of diet on chemotherapy needs further investigation in humans.

FMT with human fecal suspensions has been conducted to treat poisoning and diarrhea [133]. Furthermore, fresh or fermented fecal suspension is useful in reconstruction of the gut microbiota balance [134]. However, the application of FMT in anti-cancer treatments is still at a premature stage. A study has confirmed that transplantation of *A. muciniphila* with FMT increased the levels of IFN-γ, IL-6, and TNF-α in serum after administration of CDDP, thus enhancing CDDP’s efficacy in mice with Lewis lung cancer [135]. FMT also significantly alleviated diarrhea and intestinal mucositis in mice after FOLFOX administration [136]. With its ability to reconstruct the receptor microbiota, FMT is a promising strategy for anti-cancer treatment. Moreover, two clinical studies have confirmed that FMT enhances the anti PD-1 mAb response in patients with refractory melanoma [137,138,139]. However, FMT still faces issues such as abdominal discomfort and transmission of infections [140]. Meanwhile, FMT may transfer non-bacterial micro-organisms such as the gut virome, which raises potential safety concerns [141]. As such, it is urgent to improve the safety of FMT. Washed Microbiota Transplantation (WMT) increases the safety of fecal transplantation through employing a fecal bacteria separation system to eliminate pathogenic micro-organisms and phlogogenic factors. A clinical study confirmed that the use of WMT reduced the AE caused by FMT [142]. It is possible to envisage that FMT and WMT may undergo rapid development with the establishment of a fecal bank, a FMT registration database, and the continuous involvement of systems biology technology.

TCM has its own implications in gut microbiota interventions. It has been reported that active constituents of TCMs, such as celastrol, ephedrine, polyphenols, and apigenin exhibit anti-inflammatory effects through bacterial modulation [143]. A study has shown that *Astragalus membranaceus* inhibited the development of colon cancer in mice through inhibition of intestinal opportunistic pathogens such as *Escherichia-Shigella*, *Streptococcus*, and *Enterococcus* [144]. Gegen Qinlian Tang (GQD) is a classic TCM formula, which has been shown to enhance the anti-tumor effect of PD-1 in CRC bearing mice through increasing IFN-γ expression. Further research has revealed that the combined effect may be related to the significant enrichment of *s_uncultured_organism_g_norank_f_Bacteroidales_S24-7_group* [145]. Another example is *Poria cocos* polysaccharides (PCP), a TCM extract with various bioactivities. PCP increased the relative abundance of specific gut bacteria such as *Bifidobacterium* and *Lactobacillus* in colon cancer bearing mice, thus enhancing the efficacy of 5-FU [146]. There is also a close interaction between TCM and the gut microbiota. Several studies have found that TCMs have impacts on the composition of gut microbiota, such as *Bacteroidetes*, *Firmicutes*, and the F/B ratio (*Firmicutes/Bacteroidetes*) [147,148]. The gut microbiota can also metabolize TCMs, then convert carbohydrates and proteins into metabolites which may have beneficial or harmful effects on human health [149]. However, the use of TCMs still faces limitations such as limited understanding of the related mechanisms, difficulty in absorption, and a lack of modern scientific validation of the inherent complexity of TCMs. To address these issues, a comprehensive multi-omics platform based on gut microbiota [150] and a database related to TCM–gut microbiota research [151] can be used to characterize the active ingredients related to TCM, allowing for the eventual identification of new TCMs for novel anti-cancer strategies [149]. With the development of gut microbiota sequencing technology, fecal bacteria transplantation, precise editing of the gut microbiota, and synergistic chemotherapeutic strategies based on gut microbiota interventions are promising research areas for improving the outcomes of chemotherapy.

Taken together, immune homeostasis regulation-based gut bacterial intervention plays important roles in the sensitivity to chemotherapy. The combination of beneficial gut microbiota with chemotherapy may provide new therapeutic prospects for anti-tumor treatment.

## 6. Conclusions

The critical contributions of the gut microbiota toward human immune regulation in anti-cancer therapy have just begun to be elucidated, such as the immuno-regulatory impact of the gut microbiota on bacterial translocation, metabolite derivation, secretion of inflammatory cytokines, and so on [79]. As described in this review, the gut microbiota affect drug metabolism, immune responses, and the generation of metabolites to improve the efficacy of chemotherapy and reduce its side effects. Recent studies have focused on the discovery of effective microbes for use in interventions to improve the efficacy of chemotherapy and have made prominent achievements. Moreover, the composition and metabolic activity of the gut microbiota can be used to predict the outcome of chemotherapy [116]. However, the mechanisms driving the interactions between the gut microbiota and the immune system need further investigation. The limited availability of metagenomics and insufficiency of bacterial metabolite functional annotations restrict the development of gut functional microbiology. Meanwhile, the culture of gut anaerobes and aerobes is still a major obstacle in vitro. In addition, the safe dose of gut microbes and their negative effects are comparatively less well-understood. There are also ethical problems related to the transplantation of fecal microbiota. Future work should, therefore, aim to determine the underlying mechanism of the interactions between gut microbiota-mediated immune regulation and the response to chemotherapy. Furthermore, a combination of high-throughput sequencing, macrogenomics, and metabolomics may enhance our understanding of how to optimize the immune response, allowing for the precise regulation of pro-chemotherapy gut microbiota and, thus, providing a necessary perspective for improving the efficiency of anti-cancer treatments.

## Figures and Tables

**Table 1:** The effects of gut microbiota on the efficacy and toxicity of chemotherapy through immune regulation.

Cancer Species	Model	Drug	Gut Microbiota	Metabolites	Mechanism	Toxicity/Efficacy	References
EL4 lymphoma, MC38 colon carcinoma, and B16 melanoma	Subcutaneous injection in mice	Oxaliplatin and Cisplatin	*Ruminococcus* ↑ and *Alistipes* ↑	-	ROS production	Enhance efficacy	[114]
Metastasizing B16F10 melanomas and non-metastasizing MCA205 sarcomas	Subcutaneous injection in mice	CTX	*Lactobacillus johnsonii* ↑ and *E. hirae* ↑	-	Promotes IL-17 production by CD4 T-cells	Enhance efficacy	[111]
-	Mice	Irinotecan	*Bacteroides vulgatus* ↑ and *Clostridium ramosum* ↑	-	β-glucuronidase activates SN-38	Enhance toxicity	[120]
-	Mice	MTX	Overall abundance of gut microbiota ↓	-	Gut microbiota containsTLR2 agonist PCSK	Enhance toxicity	[121]
Mice colon cancer cell line CT-26	Subcutaneous injected in mice	FOLFOX	*Prevotella* ↑	3-Oxocholic acid ↑	Significantly improved the expression of P-EGFR/P-ERK/c-MYC and LOX	Reduce efficacy	[67]
-	Mice	Paclitaxel	*Tyzzerella* ↑, *Romboutsia* ↑ and *Turicibacter* ↑	SCFAs ↑	Increased anxiety-like behavior	Enhance toxicity	[15]
Pancreatic cancer	Induction of pancreatitis by intraperitoneal injection into the right lower quadrant of KC transgenic mice for 7 h	Gemcitabine	*Lactobacillus paracasei* ↑	-	Increase IFN-γ levels to suppress Th2 cytokine production and regulate Th1/Th2 immune balance	Enhance efficacy	[119]

## Data Availability

Not applicable.
